# Interactions of momentary thought content and subjective stress predict cortisol fluctuations in a daily life experience sampling study

**DOI:** 10.1038/s41598-018-33708-0

**Published:** 2018-10-18

**Authors:** R. Linz, T. Singer, V. Engert

**Affiliations:** Max Planck Institute for Human Cognitive and Brain Sciences, Department of Social Neuroscience, 04103 Leipzig, Germany

## Abstract

Daily life stress is an omnipresent phenomenon in modern society. Research has linked prolonged activation of the hypothalamic-pituitary-adrenal axis to psychiatric and somatic diseases. Everyday stressors substantially contribute to these health risks. Despite the notion that the physiological stress response is highly dependent on concurrent psychological processes, investigations associating diurnal cortisol levels with subjective experience have primarily focused on affective states. The impact of everyday cognitive processes including thought content has been largely neglected. To investigate this link, moment-to-moment associations of psychological experience including subjective stress, thought content and affect, and cortisol levels were assessed throughout the daily routines of 289 healthy adult participants. We found that subjective stress interacted with current thought content and affect in predicting cortisol release: more negative and future-directed thoughts were associated with higher cortisol levels after experiencing subjective stress, suggesting an increase in negative future anticipation. Concurrent cortisol rises might reflect proactive coping to adequately prepare for upcoming demands. In the absence of subjective stress, more past-directed thoughts and negative affect were associated with higher cortisol levels. These findings provide evidence for a fundamental link between thought content and daily cortisol activation, and highlight the significant contribution of thought patterns to physiological stress levels.

## Introduction

Everyday stress is omnipresent in modern society. Organisms dynamically adapt to perceived stressors through activation of the sympathetic nervous system and the hypothalamic-pituitary-adrenal (HPA) axis. Subjective-psychological processes accompany and interact with these changes in neuro-physiological systems. Importantly, chronic stress and elevated levels of the associated hormone cortisol have been linked to the development of psychiatric and somatic diseases^1,2^. Thus, a firm understanding of the factors influencing diurnal cortisol release will be critical in resolving the current epidemic of stress-related disease.

The continuous adjustment of physiological activation to appropriately meet anticipated environmental demands is referred to as allostasis^3^. While acute increases in the hormone cortisol are adaptive and indicate adequate functioning of the HPA axis in response to stressors^4^, allostatic load characterizes the ‘wear and tear’ on the body through accumulated regulatory efforts during repeated challenge^5,6^. Corroborating the strong evidence associating dysregulated stress systems with depression^7,8^, recent studies have focused on altered diurnal cortisol levels to prospectively predict the onset of depression^9^ and anxiety disorders^10^. Meta-analytic evidence further links changes in the circadian rhythm of cortisol to additional markers of adverse mental and physical health conditions^11^.

Substantial accumulation of allostatic load arises from daily-life stressors^12–14^ and the accompanying affective and cognitive processes^15,16^. In line with this notion, stress research has increasingly relied on the ambulatory assessment of cortisol^17^. Diurnal levels of cortisol collected in real-life environments have thus been linked to a variety of psychosocial variables ranging from emotional experience (e.g. self-reported stress and affect)^18^ to social contextual factors such as social support^19^, momentary loneliness and solitude^20,21^, and health-related factors including the duration and quality of sleep^22^. Regarding momentary subjective-emotional experience, a growing number of studies indicate a positive correlation of self-reported stress and cortisol in a naturalistic environment^23–25^; however some studies found no direct association (for a review see^17^). Mounting evidence for a link of negative affect and increased cortisol secretion^26–29^, and of positive affect and attenuated cortisol secretion^30^ illustrates the importance to account for concurrent subjective-emotional processes when investigating endocrine activation.

Relatively fewer studies have addressed how cognitive processes, such as particular thought patterns, impact cortisol release in daily life. A theoretical framework for such a link is provided by Brosschot and colleagues^31^. They propose repeated negative thoughts about past or future stressors to prolong the physiological stress response and thereby mediate the effects of stress on health. Rumination and worry are two prominent examples of dysfunctional thought patterns with potentially negative consequence for physical health^32^. While most work suggesting a link with cortisol was conducted in the laboratory^33,34^, only few studies directly targeting these dysfunctional cognitive styles have relied on ecological momentary assessments of cortisol and subjective experience in healthy subjects^35–38^. Among those, one study found daily averages of rumination to correlate with cortisol in a mixed sample of both depressed patients and healthy controls^35^ while another showed an association of work worries and cortisol in a sample of married couples^38^.

Moving away from pathological cognitive styles, a more recent line of research employs thought-sampling to focus on the content of an individual’s stream of thoughts. Studies of mindwandering, commonly defined as self-generated thoughts unrelated to the current task or external environment^39^, have measured thought content in three distinct dimensions targeting the temporal and social focus as well as the emotional valence of thoughts (the “cube of thought”^40^). Utilizing this thought-sampling approach, we identified moment-to-moment associations of self-generated thought content and cortisol release in a previous laboratory study: more negatively toned emotional thoughts and a pattern of more past-oriented, other-directed thoughts were associated with higher cortisol levels both at rest and after an acute laboratory stressor. A pattern of more future- and self-oriented thoughts, on the other hand, was associated with decreased overall cortisol levels^41^. These laboratory findings demonstrate a general association of specific thought content and cortisol levels.

In summary, an association between affective correlates, stress and cortisol in daily life is well established. Emerging evidence from laboratory studies also suggests a link between thought patterns and cortisol levels. However, evidence for such a link in a naturalistic environment is still lacking.

To fill this gap, the current study employed an experience sampling approach^42^ to simultaneously capture subjective experience and cortisol levels with a fine-grained temporal resolution throughout the daily routines of a large sample of healthy adult participants (N = 289, 172 women; mean ± SD: 40.6 ± 9.3 years; age range: 20–55 years). While various measures of subjective experience were captured, we placed a specific focus on mental content. By measuring participants’ subjective experience in a naturalistic environment, this approach offers high ecological validity^17^. To provide a more comprehensive investigation into daily life cortisol dynamics, we additionally explored sleep parameters and the momentary social context, both of which have previously been shown to relate to endocrine activation^21,22^.

Other than in our previous laboratory work^41,43^, thought content in the current study was not analysed in clusters determined by prior principal component analysis. Rather, each dimension of the cube of thought (valence, temporal, social) was considered separately. We employed a multi-level mixed effects model to associate cortisol levels during participants’ waking hours with momentary thought content, affect, arousal and subjective stress as markers of subjective experience. In line with prior research^26,28^, we expected to find more negative affect to be predictive of higher cortisol. We further hypothesized this association to be stronger when participants indicated that they had concurrently experienced subjective stress^29^. With respect to thought content, we hypothesized more negative and more past-oriented thoughts to be generally associated with higher levels of cortisol and, again, expected the experience of subjective stress to enhance this association. This hypothesis is in line with the preservative cognition hypothesis^31^, which suggests that dwelling on past stressors prolongs cortisol signalling. Accordingly, more future-directed thoughts were expected to predict relatively attenuated cortisol levels. Evaluative threat to the self is an especially salient aspect of psychosocial stress^44^. Therefore, regarding the social dimension of thought content, we expected more self-directed thoughts, especially after stress, to be associated with higher cortisol levels. Inversely, a less egocentric focus and thus more other-directed thoughts were expected to predict relatively attenuated cortisol levels.

## Results

### Compliance

Compliance with the sampling of saliva and subjective experience was generally satisfactory: 2672 (92.46%) of the potential 2890 cortisol probes were provided over the two sampling days, while 218 (7.54%) samples were missing or found to be empty. Among the available cortisol samples, concurrent subjective experience reports were missing or incomplete in 185 (8.79%) cases, resulting in a total of 1916 complete observations entering the final model.

### Descriptive statistics

**Figure 1:**
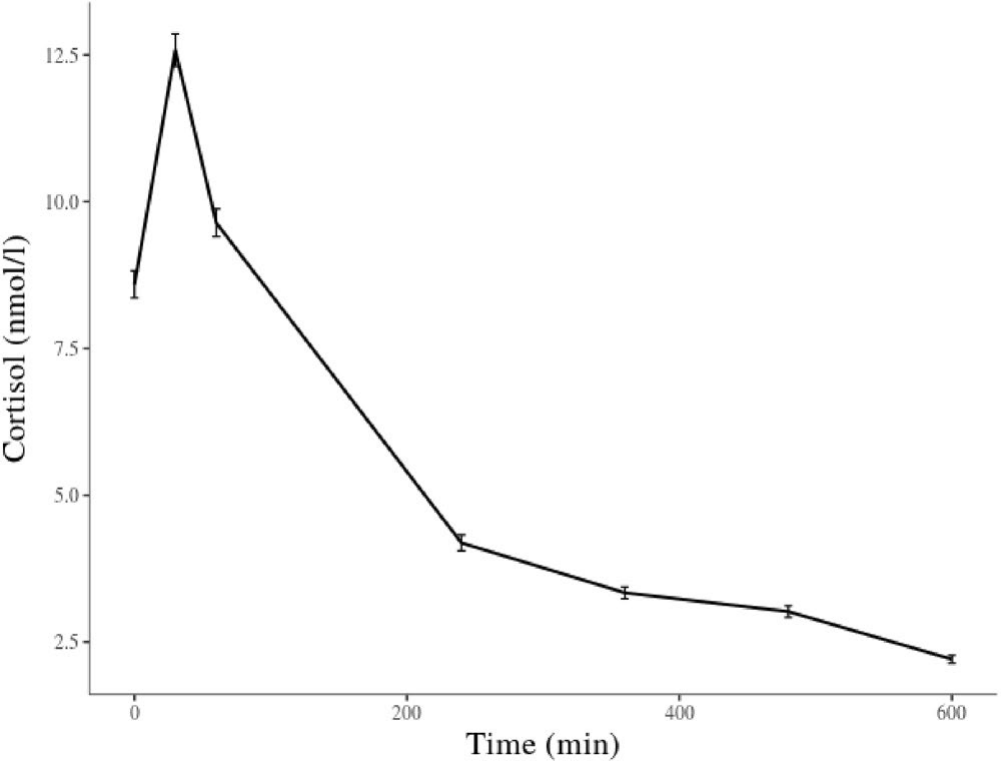
Mean observed cortisol (untransformed raw values) across the day. Time is depicted in minutes relative to awakening. Bars indicate standard errors.

Figure 1 depicts the average raw cortisol values for all measurement time-points. Average waking cortisol level in our sample was 8.59 nmol/l. As expected, after the initial steep increase, representing the cortisol awakening response, the measures throughout the day showed a gradual decrease. The average time of wakeup was 7:07 a.m. and the remaining cortisol samples corresponding with measures of subjective experience were taken on average at 11:11 a.m., 1:12 p.m., 3:13 p.m., and 5:11 p.m.

Table 1 presents the means and standard errors of the subjective experience outcomes. On average, participants displayed a tendency towards more positively-toned thought content (mean ± SE = 13.41 ± 0.07), more future-directed thought content (13.37 ± 0.07) and more positive affect (6.30 ± 0.03). Participants reported a wide range of current activities throughout their daily routines (see Supplementary Materials for details). Stressful events occurred in 20.2% of all samples.

**Table 1:** Descriptive statistics of experience sampling outcomes.

Measure	*N*	Mean (*SE*)
Affect	2912	6.30 (0.03)
Arousal	2911	4.47 (0.03)
Thoughts Valence	2910	13.41 (0.07)
Thoughts Temporal	2910	13.37 (0.07)
Thoughts Social	2910	10.65 (0.09)
Stress (no/yes) Stress magnitude Stress coping	2324/588 587 587	11.59 (0.15) 12.77 (0.16)
Company (no/yes) Closeness	1434/1478 1478	12.26 (0.15)

### Effect of subjective stress on concurrent subjective experience

Welch’s unequal variances t-tests revealed significant differences between stress and non-stress samples in several measures of subjective experience. When reporting subjective stress, participants showed a significant decrease in arousal (*t*(971.43) = −15.817, *p* < 0.001), more negative affect (*t*(766.61) = −17.143, *p* < 0.001), and more negative thoughts (*t*(829.43) = −13.641, *p* < 0.001). Temporal and social thought content did not significantly differ depending on concurrent subjective stress (both *p* values > 0.38).

### Association of subjective experience and cortisol levels

Before assessing the effects of subjective experience on cortisol levels, we fit an empty multilevel model (i.e., without predictor variables) to extract variance components of model factors and to calculate the intra class coefficient (ICC). Results indicated that 9.75% of the total variance in cortisol levels was attributed to between-individual differences. Adding ‘sampling time’ to the empty model accounted for 67.21% of the total explained variance in momentary cortisol levels and 76.96% of variance explained by the fixed factors combined, in comparison to the final full model. The remaining covariates accounted for an additional 15.23% (sex and awakening time), 1.08% (thought content), and 1.74% (affective states) of variance explained by the fixed factors compared to the final model.

**Figure 2:**
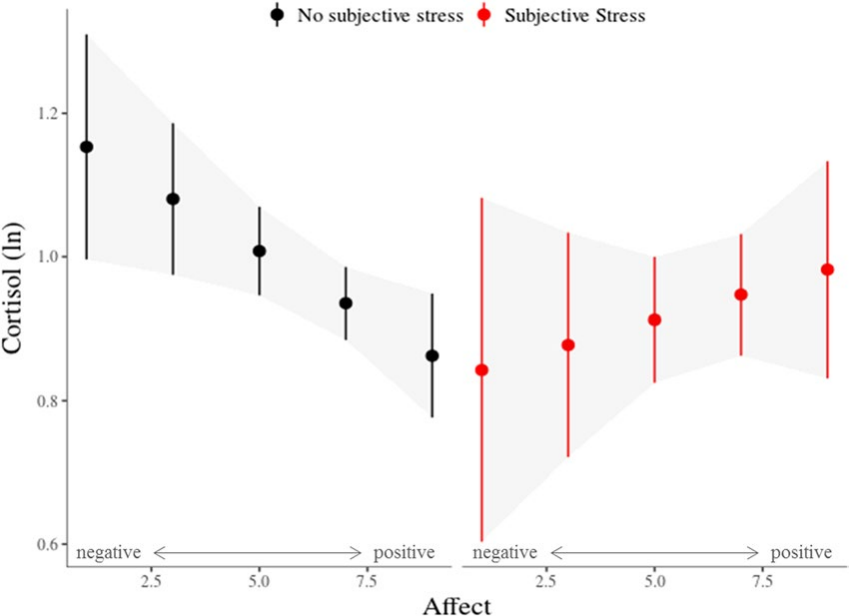
Association of cortisol (ln) and state affect (negative to positive) for samples with- and without concurrent reports of subjective stress. Bars indicate standard errors.

**Figure 3:**
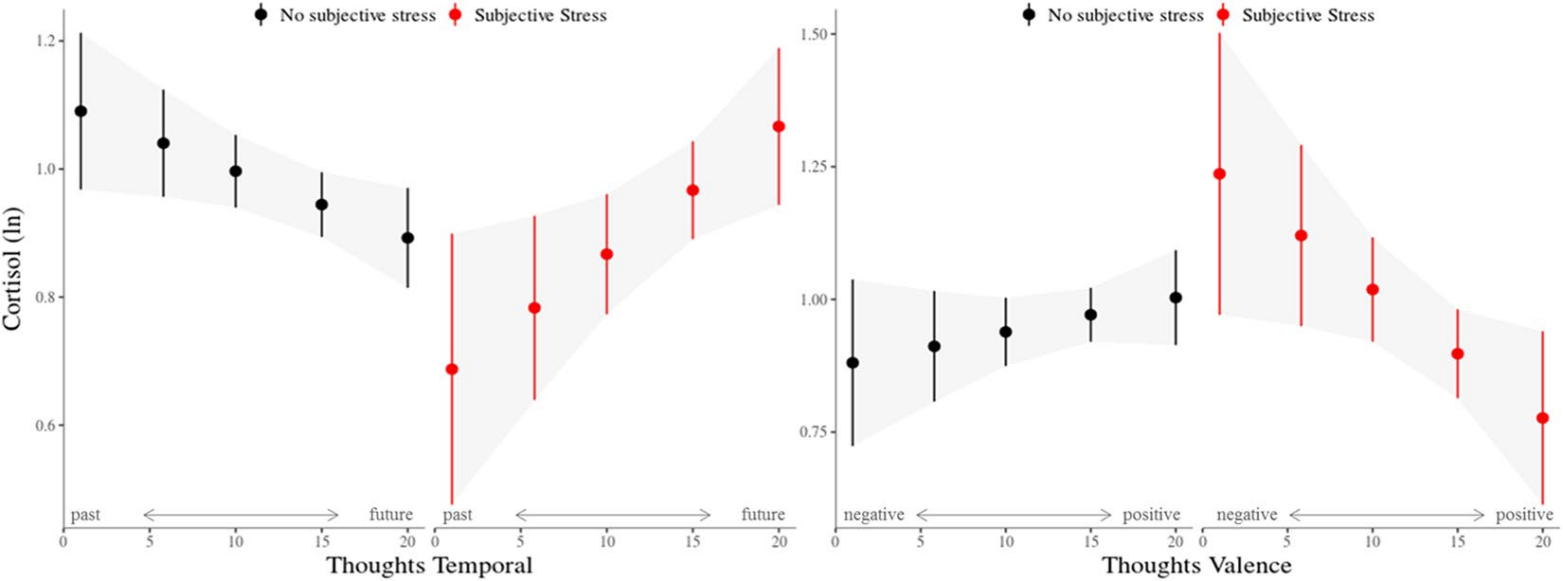
Association of cortisol (ln) and thought dimensions ‘valence’ (negative to positive) and ‘temporal’ (past-directed to future-directed) for samples with- and without concurrent reports of subjective stress. Bars indicate standard errors.

The association of cortisol levels with all available measures of subjective experience was analysed in a mixed-effects model. Table 2 shows the estimated values and margins of error (representing a 95% confidence interval) of the model parameters including all subjective experience measures, covariates (time, sex) and the contextual parameters sleep and company (i.e., momentarily being in company of someone vs. being alone). Sampling time was the strongest predictor of cortisol levels (β_10_ = −0.337, *t* = −17.27, *p* < 0.001) with longer time intervals since waking associated with lower cortisol levels. Female participants exhibited significantly lower cortisol levels than males (γ_001_ = −0.142, *t* = −3.09, *p* = 0.002). Interactions of subjective stress and affect predicted cortisol levels (π_8_ = 0.053, *t = *2.02, *p* = 0.043, see Fig. 2). Simple effects analyses revealed an effect of negative affect and cortisol only in the no-stress samples (*F* = 6.36, *p* = 0.01), with more negative affect associated with higher cortisol levels. Affect and cortisol were unrelated in the samples reporting stressful events (*F* = 1.38, *p* = 0.24). Reports of subjective stress also interacted with the temporal (π_11_ = −0.031, *t* = −3.35, *p* < 0.001) and valence (π_10_ = −0.031, *t* = −2.51, *p* = 0.01) dimensions of thought content in predicting cortisol levels (Fig. 3). The negativity of thought was associated with cortisol only after stressful experiences (*F* = 6.72, *p* < 0.01) where more negative thoughts predicted higher cortisol levels. There was no significant association in the no-stress samples (*F* = 1.39, *p* = 0.238). Past-directed thoughts predicted cortisol in the no-stress samples (*F* = 4.075, *p* = 0.044) while future-directed thoughts were predictive of cortisol after stressful experiences (*F* = 7.86, *p* < 0.01). In both cases the stronger the thoughts were rooted in one of the temporal domains, the more cortisol was released. Subjective stress did not interact with the social dimension of thought (π_12_ = −0.003, *t* = −0.62, *p* > 0.5). Momentary cortisol levels were not associated with sleep quality or sleep duration after controlling for sampling time. However, wake time predicted cortisol levels (β_03_ = −0.074, *t* = −3.642, *p* < 0.001) with higher cortisol associated with earlier waking.

**Table 2:** Model estimates for fixed effects on cortisol levels.

Fixed Effects	B (*SE*)	CI	*p*
(Intercept)	1.29 (0.159)	0.98–1.61	<0.001
Stress	−0.36 (0.175)	−0.70–−0.01	0.041
Affect	−0.04 (0.014)	−0.06–−0.01	0.010
Arousal	−0.02 (0.008)	−0.03–−0.00	0.046
Thoughts Valence	0.01 (0.006)	−0.01–0.02	0.285
Thoughts Temporal	−0.01 (0.004)	−0.02–−0.00	0.026
Thoughts Social	−0.00 (0.003)	−0.01–0.01	0.936
Sleep duration	−0.01 (0.017)	−0.04–0.03	0.648
Sleep quality	0.00 (0.004)	−0.01–0.01	0.830
Company	0.01 (0.028)	−0.04–0.07	0.661
Sex	0.14 (0.044)	0.04–0.23	0.002
Time	−0.34 (0.019)	−0.38–−0.30	<0.001
Awakening time	−0.07 (0.020)	−0.11–−0.03	<0.001
Stress*Valence	−0.03 (0.012)	−0.05–−0.01	0.012
Stress*Temporal	0.03 (0.009)	0.01–0.05	<0.001
Stress*Social	−0.00 (0.006)	−0.02–0.01	0.532
Stress*Affect	0.05 (0.026)	0.00–0.11	0.043
Stress*Arousal	0.01 (0.018)	−0.03–0.05	0.604
	**Variance**	***SD***	
**Random Effects**			
Individual (Intercept)	0.094	0.30	
Day (Intercept)	0.028	0.17	
Residual	0.248	0.49

Given that the focus of the present study was on momentary associations of cortisol with measures of subjective experience, analyses of individual differences in the diurnal cortisol rhythm will be reported elsewhere.

## Discussion

Research relating psychological concomitants of daily life to stress-related cortisol release has predominantly focused on affective states. The impact of thought content on HPA axis activation has received far less attention. In the present study, we investigated the association of diurnal cortisol levels with concurrent subjective experience in daily life, using an experience-sampling approach. In addition to relating measures of affect and subjective stress experience to diurnal cortisol levels, we sought to explore if, and how, the content of thought predicts cortisol release. We show that experiencing daily life stress increases both negative affect and the negativity of thought. However, their relation to cortisol differed depending on whether participants reported having experienced stress or not. In more detail, negative affect was associated with cortisol release only when participants did not experience a concurrent stressful event. Conversely, the negativity of thought predicted cortisol release only when subjective stress was reported. The relation of the temporal orientation of thought and cortisol was also dependent on the presence or absence of subjective stress experience: Thoughts more firmly rooted in the past were associated with higher cortisol levels in the absence of subjective stress, while more future-directed thoughts were associated with higher cortisol levels in the presence of subjective stress.

Regarding affect, we found the expected positive association between negative affect and cortisol levels, as has been shown in most prior research in ambulatory settings^25,27^. Contrary to our hypothesis however, subjective stress did not amplify this relation. Rather, no significant relation was found between momentary affect and cortisol levels after stress experience, which may in part be attributable to comparably lower statistical power in testing this association in stress-samples, which occurred much less frequently than non-stress samples. While previous studies have even proposed negative affect to mediate the effects of stress on cortisol release^29,45^, our finding suggests a general association of momentary affect and cortisol, in the absence of stress, which at the very least seems not to be enhanced after subjective stress. The existing inconsistencies among ambulatory findings might partly be due to diverging sampling designs (ranging from momentary assessments to retrospective diary methods) and different operationalization of subjective stress and affect^17^. Our data, by dissecting subjective stress, concurrent affect, and several dimensions of thought content, point towards a relation of negative affect and cortisol levels, independent of subjective stress.

With respect to cognition, we found that subjective stress modulated the interaction of thought content and cortisol regulation. Interestingly, and contrary to negative affect, the hypothesized association of cortisol and negative thought content was only observed in samples with concurrent subjective stress. Our previous laboratory findings^41^ suggested this association applied to both stress- and non-stress environments. Two main differences between our former and the present study may account for the observed inconsistencies.

First, the context and nature of the stressors fundamentally differed. The former study applied the Trier Social Stress Test (TSST^46^), a highly standardized socio-evaluative laboratory stress protocol, whereas the present study focused on daily-life experiences. While most daily life stressors are likely less pronounced than the TSST, they may be far more frequent, persistent or reoccurring, making the time of stress exposure during event sampling highly volatile^14^. Second, the character of the captured thought content differed between studies. While thought content in the former study was sampled in short intervals and during a low-demand task specifically designed to disengage attention and thus induce mindwandering, the present study sampled thought content throughout the day, over long periods of time and in various contexts. Compared to the mindwandering episodes, these daily thoughts were likely more heterogeneous, reflecting the participant’s current environment, momentary activities or social interactions. Moreover, the mindwandering pattern associated with increased baseline cortisol in our previous study comprised negatively toned *emotional* thoughts. Thus, it might rather reflect a combination of the two domains, affect and cognition, which we aimed to disentangle here. In fact, depending on the presence of stress, the current results suggest differential relations of affect and thought content with cortisol levels: While greater negative affect was generally associated with higher cortisol in the absence of subjective stress, increasingly negative thoughts were associated with higher cortisol only after stress experience. Although the measures overlap to some degree, their complementary relation to cortisol suggests the influence of distinct underlying processes, which may have different temporal dynamics. Along these lines, research has shown negative affect to be characterized by substantial short-term fluctuation^47^. The valence of thought content may be more stable over time—particularly when thoughts are still reflective of coping with the consequences of a stressor^32^ —and may thus more accurately reflect the time-point of stress exposure. Overall, the finding that negative thoughts after a stressful experience, rather than negative affect, are related to higher cortisol levels supports Brosschot’s perseverative cognition hypothesis^31^, which argues that the activation of stress systems exceeds the mere duration of a stressor and is maintained by cognitive representations of the latter.

As hypothesized, we found that the extent of past-directed thoughts predicted higher cortisol levels in the absence of stress. When reporting subjective stress, however, we found that increasingly future-oriented thoughts were associated with more pronounced cortisol levels. This later result again stands in contrast to our previous laboratory study showing future-directed mindwandering to attenuate cortisol levels, independent of prior stress exposure^41^. In addition to the explanation invoked above, the different measurement designs of stressors captured in a laboratory procedure versus an event-sampling paradigm likely played a big role in the inconsistency. While in the laboratory, mindwandering was assessed immediately following acute stress exposure, the experience sampling procedure allowed for an extended timeframe between stressor and thought sample. Experience sampling studies predicting the duration of emotions in daily life found that most emotional episodes ended within an hour of the eliciting stimulus^48^. Accordingly, the more time that passes between a stressor and the sampling of the experience, the less likely it should be that thoughts are occupied with past stressors. Indeed, our data do not show an increase in past thinking after stress experience, but rather a predominance of future thoughts irrespective of subjective stress. A second fundamental difference between laboratory-induced and experience-sampled stress was also mentioned earlier: most real-life stressors are likely to be minor^12^, characterised by less discrete onset and termination, and potentially persisting or recurring. Analyses included in our supplementary materials support this notion, showing that there was an increased likelihood of (re-)experiencing subjective stress when already having reported stress in the previous sample. It could thus be argued that the association of future thought and cortisol levels is a consequence of stress anticipation. In other words, after having experienced stress, future thought might target another stressful event yet to occur. Alternatively, the association of future thought and cortisol after experienced stress may be explained by proactive coping. Proactive coping is characterised by efforts to prevent or modify a potentially stressful event^49^. It takes place prior to anticipatory coping, and while proactive coping involves efforts to detect, prevent and proactively manage potentially occurring stressful future events, anticipatory coping targets the stressful consequences of an event that is certain to happen^50^. Stages of proactive coping involve both future simulation in order to identify potentially stressful events and initial coping efforts to prevent the likelihood of such stressors^51^. The increased release of cortisol after stress experience may thus provide the necessary energy for such proactive problem-solving activities. Potentially supporting this reasoning, a recent experience-sampling study investigating emotion regulation strategies showed that more frequent engagement in problem solving was associated with increased cortisol awakening responses^52^. Momentary problem solving, on the other hand, was associated with transient increases in cortisol levels in a subsample of participants with current internalizing disorders^52^.

Regarding health implications of the present findings, proactive coping efforts (and associated future-thinking) are primarily viewed as adaptive and have been linked to beneficial mental and physical health outcomes^50^. Several recent articles have argued against an overly simplistic view of high or rising cortisol levels as inherently adverse. Rather, they emphasize the adaptive nature and context dependency in assessing costs and benefits of acute cortisol elevation^53,54^. Along these lines, momentarily increased cortisol levels associated with future thoughts, in the present study, may well reflect adaptive coping processes. Increased cortisol may supply the ‘energetic boost’ needed to pre-empt future or terminate ongoing stressors. However, needlessly triggered or upheld stress responses—as in the case of anticipated, but non-actualised stressors—bear the risk of unnecessarily prolonging or failing to inhibit activated stress systems and may thus contribute to negative health consequences^31,55^.

Evaluative threat to the self is an especially salient aspect of psychosocial stress^44^ and it would have been conceivable that orienting thoughts away from the self, and towards others, would help buffer the stress response. However, we found no association of cortisol and the social dimension of thought; neither during stress nor in its absence. With respect to the social context, cortisol levels were unrelated to whether participants reported being alone or in company of others. While previous investigations have reported a link between momentary solitude and increased cortisol levels, in one study this association was partly moderated by the affective states correlated with being alone^21^. In another study, the association of cortisol and being alone significantly declined with age^28^, while a prior study by the same author found no association in an adult population^56^. Based on these findings suggestive of an age-dependent effect, it may have been unlikely to detect an influence of solitude on cortisol given the considerable age range of our sample (20–55 years).

Our study has some limitations. For practical reasons, we assessed cortisol and subjective experience simultaneously. However, some studies have argued for a staggered assessment to account for the dynamics of cortisol release^27^. The current study also would have benefitted from an objective verification of sampling times. Hence, the respective data have to be treated with some caution as the possibility of non-adherence-related confounding cannot be excluded^57^. Also, it would have been advantageous to increase the minimal required time interval between any oral intake and sampling as has been proposed in other studies (e.g.^18^).

Thought content in the present study was operationalized quite broadly involving three bipolar dimensions. This precluded more complex analyses that have been done in the past, such as PCA^40,41^, and limits the precision with which we were able to draw conclusions about the specific nature of thought. It would be interesting to know whether past thoughts are predominantly ruminative in quality or whether future thoughts imply worry. Not critical to our results, but certainly of interest, is to what extent our thought samples reflected mindwandering episodes (i.e., were mainly task-unrelated and self-generated), or if they were predominantly driven by the external environment. Considering that mindwandering episodes occur frequently in daily life, occupying an estimated 25% to 50% of our waking time^58,59^, certainly a fair share of the thoughts we captured here overlapped with the mindwandering characteristics of being unrelated to current task or environment. Future studies may wish to examine how thought content in a naturalistic environment translates to the frequency and content of mindwandering in the laboratory. This would be particularly fruitful considering the ongoing debate about the costs and benefits of the wandering mind^60^.

The major objective of our study was to explore the role of thought content and its interaction with experienced stress in predicting HPA axis activity in daily life. Building upon previous laboratory studies^41,61^, we examined thought-body correlations in an ecologically valid setting. We show that over a wide range of activities and contexts, throughout daily life, the content of thought is associated with cortisol release. When exploring everyday subjective experience, not only affective but also cognitive measures should be considered to further our understanding of how psychological processes shape our physiological stress levels. As stressful episodes are an inevitable part of modern life, learning to be aware and more actively control our daily thoughts may be an important first step in regulating excessive activation of the HPA axis. As a next step, within the scope of the *ReSource Project*, we will investigate whether distinct mental training modules targeting the plasticity of mental content indeed have the capacity to change our thought-body correlations towards the aim of reducing stress and fostering well-being.

## Methods

### Participants

Participants attended the baseline measurement of the *ReSource Project*, a large-scale, longitudinal, mental training study conducted in Leipzig and Berlin^62^. Volunteers were recruited in winter 2012/2013 and underwent a comprehensive mental health diagnostic interview with a trained clinical psychologist. The interview included a computer-assisted German version of the structured Clinical Interview for DSM-IV Axis-I disorders, the SCID-I DIA-X^64^, and a personal interview for Axis-II disorders, the SCID-II^64,65^. Volunteers were excluded if they fulfilled criteria for an Axis-I disorder within the past two years, or for schizophrenia, psychotic disorder, bipolar disorder, substance dependency or an Axis-II disorder at any time in their life. Volunteers taking medication influencing the HPA axis were also excluded. Details of the multistep recruitment procedure, the complete list of inclusion/exclusion criteria and the final sample description of the *ReSource Project* can be found in Singer *et al*.^62^. Of the total 332 participants enrolled in the *ReSource Project*, 289 (172 women, mean ± SD = 40.6 ± 9.3 years, 20–55 years, all Caucasian) provided data for the present study, which was conducted at the baseline testing time-point before any mental training had taken place. The missing 43 participants either dropped out of the study (*N* = 22, 12 women), were excluded from the study (*N* = 18, 12 women), were repeatedly unavailable or provided insufficient data for the experience sampling (*N* = 3, 1 woman). Reasons for drop-out were time constraints (*N* = 17, 8 women) and discomfort with the study or experiments (*N* = 5, 4 women). Reasons for exclusion were medical (*N* = 12, 8 women) or prior mental training experience (*N* = 6, 4 woman). A higher education degree (e.g. university) was held by 65.4% of participants, whereas 24.7% held a higher education entrance qualification (e.g., high-school diploma) and 9.89% received lower secondary education. Participants’ median annual household net income was €30,000 (mean ± SD = 35,832 ± 20,493). The *ReSource Project* was registered with the Protocol Registration System of ClinicalTrial.gov on April 16^th^, 2013 under the title “Plasticity of the Compassionate Brain” (Identifier NCT01833104). It was approved by the Research Ethics Boards of Leipzig University (ethic number: 376/12-ff) and Humboldt University Berlin (ethic numbers: 2013-20, 2013-29, 2014-10). All methods were performed in accordance with the protocols mentioned above. Participants gave written informed consent, could withdraw from the study at any time and were financially compensated.

### Procedure

Participants were advised to choose two regular consecutive weekdays, representative of their daily life routines, for saliva- and experience sampling (e.g. Mondays/Tuesdays, Wednesdays/Thursdays or Thursdays/Fridays, depending on their availability). All experience sampling data was collected in a standardized manner using mobile devices equipped with a customized software application that reminded participants to take each sample at the designated time of day. Before data collection, participants received an introductory training to ensure proficiency in handling the mobile device and self-administering saliva samples.

**Figure 4:**
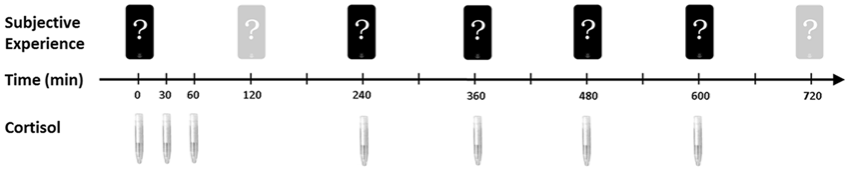
Time schedules of salivary cortisol and experience samples relative to wakeup.

In detail, sampling times for saliva were immediately upon free awakening (while still in bed), and 30, 60, 240, 360, 480 and 600 minutes thereafter. Experience sampling questionnaires were answered upon waking and in intervals of 120 minutes until 12 hours after wake up (i.e., 120, 240, 360, 480, 600 and 720 minutes, see Fig. 4). The awakening probe, as well as probes at 240, 360, 480 and 600 minutes overlapped with the cortisol sampling schedule. Because we focus only on concurrent samples of subjective experience and cortisol, the samples taken at 30 and 60 minutes after waking (which capture the cortisol awakening response) were not included in the current analysis. Sampling times during the day had a margin of fluctuation (+/−15 min of the fixed interval) to avoid complete predictability.

### Measures

#### Salivary cortisol

Saliva was sampled with Salivette collection devices (Sarstedt, Nümbrecht, Germany). Participants were instructed to place the collection swabs in their mouths and to refrain from chewing for 2 min. They were asked to take nothing by mouth other than water, to refrain from brushing their teeth for 10 minutes, and from smoking for 30 minutes prior to sampling. If deviating from this guideline, they were asked to thoroughly rinse their mouth with water before taking a sample. Participants otherwise followed their normal daily routine. To control for confounding effects of female hormonal status on cortisol^66^, hormonal status was assessed via self-report prior to the sampling days (i.e., no menstrual cycle because of menopause or polycystic ovary syndrome, hormonal contraceptives, natural menstrual cycle, male). Salivettes were stored in participants’ freezers until returned to the laboratory where they were stored at −30 °C until assay (Department of Biological and Clinical Psychology, University of Trier, Germany). Cortisol levels (expressed in nmol/l) were determined using a time-resolved fluorescence immunoassay with intra-/interassay variabilities of <10%/12%^67^.

#### Subjective experience

Throughout the day, when probed, participants provided information on their momentary subjective experience including thought content, affect and arousal, and the occurrence of subjective stress. Further contextual information included the current activity and whether participants were alone or in the company of others when sampling.

Thought content: Participants indicated current thought content along the three dimensions of the cube of thought^40^. Unlike previous studies, which used PCA to extract patterns of thought content based on covariance of several single content items^40,41^, in the current study we operationalized thought content along only three dimensions with opposing poles (e.g., negative to positive), precluding the previously used approach. The thought dimensions were represented by three visual analogue scales (valence, temporal and social), each ranging from 1–20 with written anchors at each pole (“Please categorize your current thoughts”). The valence scale was anchored by ‘negative’ and ‘positive’, the temporal scale by ‘past-oriented’ and ‘future-oriented’ and the social scale by ‘self-oriented’ and ‘other-oriented’.

Affect and arousal: Measures of momentary affect and arousal were obtained using the Affect Grid, a single item scale assessing the dimensions pleasure-displeasure and arousal-sleepiness (both on a scale from 1–9). The Affect Grid has adequate reliability, convergent and discriminant validity^68^.

Subjective stress: At each sampling point, participants reported on the occurrence of subjective stress in a yes/no format. If they had experienced a stressful event since taking their last sample, they additionally indicated how stressed they felt and how well they were able to cope with the stressor (both on visual analog scales ranging from 1–20). Since these stress characteristics were contingent on reporting a subjective stress experience, they were only available in a subsample and were thus analysed separately (see Supplementary Materials).

Company: Participants reported on whether they were currently alone or in company of others when completing a probe. If with others, they additionally indicated how close they felt to their company (on a visual analog scale ranging from 1–20).

Sleep: Duration (in hours) and quality of sleep (on a visual analog scale ranging from 1–20) was reported immediately upon awakening in parallel to taking the first Salivette of the day.

### Statistical analysis

To assess the moment-to-moment associations of cortisol and self-report data, a multi-level linear mixed effects model was fitted to the hierarchical data structure: Samples (level 1) within days (level 2) were nested within individuals (level 3). The model integrates all available parallel measures of cortisol and subjective experience (at 240, 360, 480 and 600 minutes) and includes day- and person-specific fixed and random factors.

Initially, an empty multi-level model including only a random intercept for subject was fitted to the data in order to assess the intra class coefficient (ICC). For the final model, predictors entering on the sample level (level 1) were the cortisol sampling time relative to awakening (time), the occurrence of subjective stress (stress), affect and arousal, the respective thought content dimensions (valence, temporal, social), whether participants were currently with someone or alone (company) and the interaction terms of subjective stress with affect, arousal and thought content (stress*affect, stress*arousal, stress*valence, stress*temporal, stress*social). Predictors corresponding to each day (level 2) were sleep duration, sleep quality, and awakening time, nested within individuals (level 3) with a fixed factor for sex. A random intercept for subjects and days nested into subjects as well as random slopes for stress and time were included to increase model fit. The final model including all fixed and random effects is specified below in Raudenbush and Bryk^69^ notation:

1 $$\begin{array}{ccc}{{\rm{Y}}}_{{\bf{s}}{\rm{di}}} & = & {{\rm{\pi }}}_{{\rm{0di}}}+{{\rm{\pi }}}_{{\rm{1di}}}({\rm{time}})+{{\rm{\pi }}}_{{\rm{2di}}}({\rm{stress}})+{{\rm{\pi }}}_{{\rm{3di}}}({\rm{affect}})+{{\rm{\pi }}}_{{\rm{4di}}}({\rm{arousal}})\\  &  & +\,{{\rm{\pi }}}_{{\rm{5di}}}({\rm{valence}})+{{\rm{\pi }}}_{{\rm{6di}}}({\rm{temporal}})+{{\rm{\pi }}}_{{\rm{7di}}}({\rm{social}})+{{\rm{\pi }}}_{{\rm{8di}}}({\rm{stress}}\ast {\rm{affect}})\\  &  & +\,{{\rm{\pi }}}_{{\rm{9di}}}({\rm{stress}}\ast {\rm{arousal}})+{{\rm{\pi }}}_{{\rm{10di}}}({\rm{stress}}\ast {\rm{valence}})+{{\rm{\pi }}}_{{\rm{11di}}}({\rm{stress}}\ast {\rm{temporal}})\\  &  & +\,{{\rm{\pi }}}_{{\rm{12di}}}({\rm{stress}}\ast {\rm{social}})+{{\rm{\pi }}}_{{\rm{13di}}}({\rm{company}})+{{\rm{e}}}_{{\rm{tdi}}}\end{array}$$

2 $$\begin{array}{c}{{\rm{\pi }}}_{{\rm{0di}}}={{\rm{\beta }}}_{{\rm{00i}}}+{{\rm{\beta }}}_{{\rm{01i}}}({\rm{sleep}}\,{\rm{duration}})+{{\rm{\beta }}}_{{\rm{02i}}}({\rm{sleep}}\,{\rm{quality}})+{{\rm{\beta }}}_{{\rm{03i}}}({\rm{awakening}}\,{\rm{time}})+{{\rm{u}}}_{{\rm{0di}}}\\ {{\rm{\pi }}}_{{\rm{1di}}}={{\rm{\beta }}}_{{\rm{10i}}}+{{\rm{u}}}_{{\rm{1di}}}\\ {{\rm{\pi }}}_{{\rm{2di}}}={{\rm{\beta }}}_{{\rm{20i}}}+{{\rm{u}}}_{{\rm{2di}}}\end{array}$$

3 $$\begin{array}{c}{{\rm{\beta }}}_{{\rm{00i}}}={{\rm{\gamma }}}_{{\rm{000}}}+{{\rm{\gamma }}}_{{\rm{001}}}({\rm{sex}})+{{\rm{r}}}_{{\rm{00i}}}\\ {{\rm{\beta }}}_{{\rm{10i}}}={{\rm{\gamma }}}_{{\rm{100}}}+{{\rm{r}}}_{{\rm{10i}}}\\ {{\rm{\beta }}}_{{\rm{20i}}}={{\rm{\gamma }}}_{{\rm{200}}}+{{\rm{r}}}_{{\rm{20i}}}\end{array}$$

Cortisol levels for a sample *s* on day *d* for subject *i* (Level 1) were predicted by the intercept (π_0di_), the time of the sample relative to waking up (π_1di_), the occurrence of subjective stress (π_2di_), company (π_13di_) and the affective and cognitive measures of subjective experience (π_3di_ − π_7di_) as well as their respective interactions with subjective stress (π_8di_ − π_12di_).

On level 2, β_01i_ − β_03i_ describe the day-specific factors entering the model (sleep duration, sleep quality, awakening time), and u_0di_, u_1di_, u_2di_, are the respective random effects of intercept, time and stress, which account for variation within individuals across days.

On level 3, γ_001_ reflects the individuals’ sex, while r_00i_, r_10i_ and r_20i_ are the random factors accounting for individual differences in the intercept, time and stress.

The final model included only a linear time term. Adding a quadratic time term to the model yielded only very minimal increases in explained variance, particularly in comparison to the linear term, did not change the pattern of results and was thus not included. Likewise, the additional person-specific covariates age and hormonal status were not included in the final model since they had no significant effect on cortisol and did not improve the relative model quality.

Raw cortisol values were ln-transformed before analysis to account for positive skew (however analyses with raw values yielded negligible differences with respect to the estimated effects). The model’s residuals displayed satisfactory approximation to normal distribution and calculation of variance inflation factors indicated moderate, but tenable levels of multicollinearity of the model factors^70^. Estimates reported are restricted maximum likelihood marginal estimates using an unstructured covariance structure.

Several additional analyses were conducted for conditional subsamples. The respective models were derived from the main model specified above and fit to assess a) the effects of stressor characteristics on cortisol levels in the samples reporting subjective stress, and b) the effects of closeness of one’s company for the samples in which participants reported being in company of others (see Supplementary Materials for details).

Finally, to examine whether thought content differed in samples with reported subjective stress, as compared to non-stress samples, t-tests were calculated for measures of subjective experience. We used Welch’s t-tests for these independent samples with unequal variances and unequal sample sizes^71^. Variance components explained by the fixed and random factors were examined by stepwise addition of factors of interest to an empty model only containing random factors and subsequent comparison to the final model^72^. All analyses were performed using *R* version 3.3.2 (R Core Team, 2016) and the packages *lme4*, *car and Mumin* were used to fit models and obtain p-values and measures of explained variance. Significance was set to a level of p < 0.05.

## Electronic supplementary material

Supplementary Materials

## Data Availability

The datasets generated and/or analysed during the current study are not publicly available due to ongoing analyses in the context of the large-scale *ReSource Project*. The data are available on reasonable request.
