# Supplementary material for: Interactions of momentary thought content and subjective stress predict cortisol fluctuations in a daily life experience sampling study
Source: Sci Rep. 2018 Oct 18;8:15462. doi: 10.1038/s41598-018-33708-0 (PMC6193976; doi:10.1038/s41598-018-33708-0)
Supplement: Supplementary file 1 — Supplementary Materials [file 41598_2018_33708_MOESM1_ESM.pdf]

Supplementary Materials for

Interactions of momentary thought content and subjective stress predict cortisol fluctuations in a daily life experience sampling study

Linz, R.*, Singer, T.†, Engert, V.†

Max Planck Institute for Human Cognitive and Brain Sciences, Department of Social Neuroscience, 04103 Leipzig, Germany

†Joint last authors

*Corresponding author: Roman Linz

Supplementary statistical analysis

Exploratory analyses. To assess frequencies of specific thought patterns, the three-dimensional cube of thought was divided into subfields most closely resembling thought clusters of rumination (negative, self- and past-directed thought) and worry (negative, self- and future-directed thought). These subfields constituted thoughts that combined scores in the outmost half of a respective pole (e.g., negative pole) of the respective thought dimensions (e.g., valence dimension).

Effects of stressor characteristics on cortisol levels in samples with reports of subjective stress. To assess the association of both the stress magnitude and the success of coping with the stressor with cortisol levels, the following model (Model 2) was derived from the main model and fit to a conditional subsample of the dataset (i.e., all samples reporting subjective stress):

Level 1: $Y_{sdi} = \pi_{0di} + \pi_{1di}$ (time) $+ \pi_{2di}$ (stress magnitude) $+ \pi_{3di}$ (stress coping) $+ \pi_{4di}$ (stress magnitude*stress coping) $+ e_{tdi}$

Level 2: $\pi_{0di} = \beta_{00i} + \beta_{02i}$ (awakening time) $+ u_{0di}$

Level 3: $\beta_{00i} = \gamma_{000} + \gamma_{001}$ (sex) $+ r_{00i}$

Effects of closeness of one's company on cortisol levels. For those samples in which participants reported being in company of others, the association of cortisol levels with the closeness of one's company was assessed with the following model (Model 3):

Level 1: $Y_{sdi} = \pi_{0di} + \pi_{1di}\,(\text{time}) + \pi_{2di}\,(\text{closeness}) + e_{tdi}$

Level 2: $\pi_{0di} = \beta_{00i} + \beta_{02i}\,(\text{awakening time}) + u_{0di}$

Level 3: $\beta_{00i} = \gamma_{000} + \gamma_{001}\,(\text{sex}) + r_{00i}$

Lag analysis. To explore the association between reports of stress in a current sample with reports of stress in the following sample, we used a Pearson's Chi-squared test with Yates' continuity correction for a 2x2 table with the current and lagged report of subjective stress as variables of interest.

Supplementary results

Descriptive *a*nalysis of reported activities revealed a wide range of sampled activities throughout the daily routines of participants. Most frequently, these activities were work-related (>58%), followed by being on the computer/online (14.8%), commuting (7.8%), and relaxing or resting (4.9%).

Analysis of frequencies of thought clusters (operationalized as subfields of the three-dimensional cube of thought) revealed 10 cases (0.2%) of potentially ruminative thought content (marked by the conjunction of pronounced negative, self- and past-directed content) and 23 cases (0.4%) of potentially worrysome thought content (marked by the conjunction of pronounced negative, self- and future-directed content).

Table S1 shows the results for Model 2. In those samples which reported subjective stress, neither the magnitude of the stressor ($p > .9$) nor the coping success ($p > .7$) or their interaction ($p > .8$) were related to cortisol levels.

Table S2 shows the results for Model 3. In those cases in which participants reported being in company of others, the closeness they felt toward this company was not significantly associated with cortisol levels ($p = 0.214$).

Pearson's Chi-squared test revealed that the likelihood of reporting stress in a sample significantly differed depending on whether stress was reported in the previous sample. Reporting stress was more likely when having reported stress in the previous sample (43%) as opposed to reporting stress when not having reported stress in the previous sample (13,3%), $\chi^2(1, N = 2222) = 207.1$, $p < 0.001$; odds ratio = 4.93.

Table S1. Model 2 estimates for fixed effects on cortisol levels.

	B (*SE*)	*t*	*p*
Fixed Effects			
(Intercept)	1.330 (0.341)	3.89	≤0.001
Stress magnitude	-0.002 (0.023)	-0.08	>0.900
Stress coping	-0.007 (0.022)	-0.30	>0.700
Stress magnitude*coping	>0.001 (0.001)	0.21	>0.800
Sex	-0.143 (0.066)	-2.18	0.031
Time	-0.373 (0.041)	-9.08	≤0.001
Awakening time	>-0.001 (0.001)	-1.92	0.052

Table S1. Cortisol levels were ln transformed. Model based on 457 observations.

Table S2. Model 3 estimates for fixed effects on cortisol levels.

	B (SE)	*t*	*p*
Fixed Effects			
(Intercept)	1.311 (0.094)	13.92	≤0.001
Closeness of company	-0.004 (0.004)	-1.24	0.214
Sex	-0.149 (0.056)	-2.66	0.008
Time	-0.318 (0.027)	-11.91	≤0.001
Awakening time	>-0.001 (0.001)	-2.53	0.012

Table S2. Cortisol levels were ln transformed. Model based on 1105 observations.
